# Reply: Somatic mutations are present in all members of the AKT family in endometrial carcinoma

**DOI:** 10.1038/sj.bjc.6605302

**Published:** 2009-09-08

**Authors:** K Shoji, K Oda, S Nakagawa, S Hosokawa, G Nagae, Y Uehara, K Sone, Y Miyamoto, H Hiraike, O Hiraike-Wada, T Nei, K Kawana, H Kuramoto, H Aburatani, T Yano, Y Taketani

**Affiliations:** 1 Department of Obstetrics and Gynecology, The University of Tokyo, 7-3-1 Hongo B unkyo-ku, Tokyo, Japan; 2 Genome Science Division, Research Center for Advanced Science and Technology, The University of Tokyo, 4-6-1 Komaba Meguro-ku, Tokyo, Japan; 3 Department of Clinical Cytology, Kitasato University Graduate School of Medical Sciences, Kitasato 1-15-1 Sagamihara-shi, Kanagawa, Japan

**Sir,**

We appreciate the attention given by Drs Dutt, Salvesen, Greulich, Sellers, Beroukhim and Meyerson to our recent publication ‘The oncogenic mutation in the pleckstrin homology domain of AKT1 in endometrial carcinomas’ (Shoji *et al*, 2009). In this article, we report a 2% mutational frequency of *AKT1* (E17K) among 101 endometrial carcinomas. We also described that these two *AKT1* mutant tumours do not possess any mutations in *PIK3CA*, *PTEN* and *K-Ras*. Our report has proposed two issues to be clarified: (1) Are there any ‘oncogenic’ mutations in other AKT family members in endometrial carcinomas? (2) Are all the AKT family mutations mutually exclusive with other PI3 kinase-AKT-activating mutations?

Including the data in their earlier report, Dutt *et al* (2008) revealed mutations in *AKT2* and *AKT3*, as well as in *AKT1*. As for *AKT1* (E17K) mutations, their data and our data are compatible, showing that *AKT1* mutations were detected in 2% of the endometrioid subtype in endometrial cancer. Compared with the accumulative data in *AKT1* (E17K) mutations, mutations in *AKT2* and *AKT3* are not well characterised. Carpten *et al* (2007) and Kim *et al* (2008) reported no E17K mutations in *AKT2* and *AKT3* in breast, colorectal, gastric, hepatocellular, lung carcinomas and acute leukaemias. Davies *et al* (2008) first reported *AKT3* (E17K) mutations in melanoma at 1.5% frequency. Parsons *et al* (2005) found two mutations of *AKT2* (S302G and R371H) in 204 colorectal cancer samples, and Soung *et al* (2006) reported one missense mutation (A377V) and two possible splice-site mutations in intron 11 of *AKT2* in gastric and lung adenocarcinoma. However, the physiological role of *AKT2* mutations, including those (D399N, R368C and D32H) reported by Dutt *et al* (2008), has not been validated yet. In addition, the *AKT3* (E438D) mutation has not been reported in any type of tumours. It is important to clarify whether these rare AKT2/3 mutations cause an oncogenic effect in cancer.

We previously reported that *PIK3CA* mutations frequently coexist with mutations in *PTEN* and/or *KRAS*, and suggested that the *PIK3CA* mutation might require another upstream input to fully activate the PI3K kinase–AKT pathway (Oda *et al*, 2005, 2008). Their data of coexistent mutations in *AKT1* (E17K) and *KRAS* (G13D) suggest that the *KRAS* mutation alone is insufficient for a full activation of the PI3 kinase–AKT pathway. Their data are not inconsistent with the hypothesis that the *AKT1* (E17K) mutation and the *PIK3CA* mutation are mutually exclusive. The coexistent mutations of *AKT3* and *PIK3CA* in one sample suggest that some types of *AKT* mutations (non-E17K) might coexist with *PIK3CA* mutations to enhance the activation of the PI3 kinase–AKT pathway. Further analyses are necessary to clarify the frequency of coexistent mutations in *AKT1* (E17K), *AKT2/3* and other mutations.

Their current data and our data suggest that the PI3 kinase–AKT pathway is prevalently activated in endometrial cancer through various genetic alterations and their combinations. Further analyses in *AKT*, *PIK3CA* and other PI3 kinase-AKT-related genes would be helpful to comprehensively understand the mechanism of activation in this pathway and to use PI3K-targeted therapies in various types of cancer.

We thank Dr Dutt and colleagues for their recognition of our work.

## References

[bib1] Carpten JD, Faber AL, Horn C, Donoho GP, Briggs SL, Robbins CM, Hostetter G, Boguslawski S, Moses TY, Savage S, Uhlik M, Lin A, Du J, Qian YW, Zeckner DJ, Tucker-Kellogg G, Touchman J, Patel K, Mousses S, Bittner M, Schevitz R, Lai MH, Blanchard KL, Thomas JE (2007) A transforming mutation in the pleckstrin homology domain of AKT1 in cancer. Nature 448: 439–444 17611497 10.1038/nature05933

[bib2] Davies MA, Stemke Hale K, Tellez C, Calderone TL, Deng W, Prieto VG, Lazar AJ, Gershenwald JE, Mills GB (2008) A novel AKT3 mutation in melanoma tumours cell lines. Br J Cancer 99: 1265–1268 18813315 10.1038/sj.bjc.6604637 PMC2570525

[bib3] Dutt A, Salvesen HB, Chen TH, Ramos AH, Onofrio RC, Hatton C, Nicoletti R, Winckler W, Grewal R, Hanna M, Wyhs N, Ziaugra L, Richter DJ, Trovik J, Engelsen IB, Stefansson IM, Fennell T, Cibulskis K, Zody MC, Akslen LA, Gabriel S, Wong KK, Sellers WR, Meyerson M, Greulich H (2008) Drug-sensitive FGFR2 mutations in endometrial carcinoma. Proc Natl Acad Sci USA 105: 8713–9717 18552176 10.1073/pnas.0803379105 PMC2438391

[bib4] Kim MS, Jeong EG, Yoo NJ, Lee SH (2008) Mutational analysis of oncogenic AKT E17K mutation in common solid cancers acute leukaemias. Br J Cancer 98: 1533–1535 18392055 10.1038/sj.bjc.6604212 PMC2391109

[bib5] Oda K, Stokoe D, Taketani Y, McCormick F (2005) High frequency of coexistent mutations of PIK3CA PTEN genes in endometrial carcinoma. Cancer Res 65: 10669–10673 16322209 10.1158/0008-5472.CAN-05-2620

[bib6] Oda K, Okada J, Timmerman L, Rodriguez Viciana P, Stokoe D, Shoji K, Taketani Y, Kuramoto H, Knight ZA, Shokat KM, McCormick F (2008) PIK3CA cooperates with other phosphatidylinositol 3′-kinase pathway mutations to effect oncogenic transformation. Cancer Res 68: 8127–8136 18829572 10.1158/0008-5472.CAN-08-0755

[bib7] Parsons DW, Wang TL, Samuels Y, Bardelli A, Cummins JM, DeLong L, Silliman N, Ptak J, Szabo S, Willson JK, Markowitz S, Kinzler KW, Vogelstein B, Lengauer C, Velculescu VE (2005) Colorectal cancer: mutations in a signalling pathway. Nature 436: 792 16094359 10.1038/436792a

[bib8] Shoji K, Oda K, Nakagawa S, Hosokawa S, Nagae G, Uehara Y, Sone K, Miyamoto Y, Hiraike H, Hiraike-Wada O, Nei T, Kawana K, Kuramoto H, Aburatani H, Yano T, Taketani Y (2009) The oncogenic mutation in the pleckstrin homology domain of AKT1 in endometrial carcinomas. Br J Cancer 101: 145–148 19491896 10.1038/sj.bjc.6605109 PMC2713716

[bib9] Soung YH, Lee JW, Nam SW, Lee JY, Yoo NJ, Lee SH (2006) Mutational analysis of AKT1 AKT AKT3 genes in common human carcinomas. Oncology 70: 285–289 17047397 10.1159/000096289

